# The Effect of Direction on Cursor Moving Kinematics

**DOI:** 10.3390/s120201919

**Published:** 2012-02-10

**Authors:** Ling-Fu Meng, Hsin-Yung Chen, Chiu-Ping Lu, Ming-Chung Chen, Chi-Nung Chu

**Affiliations:** 1 Department of Occupational Therapy, Institute of Clinical Behavioral Science, Chang Gung University, No. 259, Wen-Hwa 1st Road, Kwei-Shan, Taoyuan 333, Taiwan; E-Mails: lfmeng@mail.cgu.edu.tw (L.-F.M.); glorialu1229@hotmail.com (C.-P.L.); 2 Department of Special Education, National Chiayi University, No. 300, Syuefu Road, Chiayi 600, Taiwan; E-Mail: ming@chen.twmail.cc; 3 Department of Information Management, China University of Technology, No. 56, Section 3, Xinglong Road, Wunshan District, Taipei 116, Taiwan; E-Mail: nung@cute.edu.tw

**Keywords:** cursor moving direction, kinematics, laterality

## Abstract

There have been only few studies to substantiate the kinematic characteristics of cursor movement. In this study, a quantitative experimental research method was used to explore the effect of moving direction on the kinematics of cursor movement in 24 typical young persons using our previously developed computerized measuring program. The results of multiple one way repeated measures ANOVAs and *post hoc* LSD tests demonstrated that the moving direction had effects on average velocity, movement time, movement unit and peak velocity. Moving leftward showed better efficiency than moving rightward, upward and downward from the kinematic evidences such as velocity, movement unit and time. Moreover, the unique pattern of the power spectral density (PSD) of velocity (strategy for power application) explained why the smoothness was still maintained while moving leftward even under an unstable situation with larger momentum. Moreover, the information from this cursor moving study can guide us to relocate the toolbars and icons in the window interface, especially for individuals with physical disabilities whose performances are easily interrupted while controlling the cursor in specific directions.

## 1. Introduction

Only a few studies delineate the effect of cursor moving direction on the kinematic parameters [1–6]. Exploring this information can expand and enhance our basic knowledge on matters of laterality from the aspect of the human-computer interaction. Moreover, this laterality characteristic is helpful to guide us to modify computer accessibility, especially for persons with special needs [5,7–10]. For example, if a person cannot perform smoothly while moving the cursor to the left, one possible solution is to position the icons or toolbars on the right hand of the screen to diminish the need to move leftward as a modified access method. Therefore, besides knowing and describing the basic phenomena, the study of cursor movement laterality can also provide information with clinical implications. In 2007, we initiated a study to explore the cursor dragging kinematics (not cursor movement) in healthy participants. The directional effect on the parameter of movement time and movement units was found and the leftward dragging movement showed an obvious advantage when comparing with other directions [10]. Based on the results of cursor dragging kinematics, it is believed that further investigation in cursor movement would provide comprehensive information about computer access for clinical applications. In this study, we will report the characteristics of cursor movement kinematics in healthy subjects.

Some studies related to the effect of direction on cursor movement have been conducted (Table 1). Most studies demonstrated that moving towards the left direction is more efficient than moving towards other directions. In 1998, Phillips *et al.* instructed the participants to use the Accupoint to position the cursor and found that the duration time of leftwards (mean = 840.9 ms) and rightwards (mean = 954.6 ms) movements were longer than cursor moving for vertical movements (mean = 706.1 ms) [1]. In 2001, Phillips and Triggs further substantiated the directional effects on the kinematic characteristics of cursor moving [2]. They found moving rightward was slower than moving towards other directions. According to the findings of Phillips *et al.*, cursor moving performance was affected by the moving direction. In 2003, Phillips *et al.* initiated a new study to address the impact of the cursor’s orientation and target’s size on its positioning (cursor moved to the target), the cursor was an arrow pointing to the upper left or upper right of the screen and the circular target diameter was 4, 8, or 16 mm [3]. Twelve participants were required to move the cursor rightward or leftward towards the target on a computer screen. Under the situation without considering the effect of the cursor arrow direction and the size of target, the result showed that moving leftward demonstrated better performance in reaction time, movement time and accuracy when compared with moving rightwards.

In 2007, Thompson *et al.* found that rightward and leftward movements took less time than upward and downward movements. They further explained that the lower inertial requirements of only a single joint (*i.e.*, the elbow) while moving leftwards and rightwards can make performance more efficient [4]. Moreover, moving towards the right or left also reached higher peak velocities than vertical movements. Thompson *et al.* inferred that the higher peak velocities might cause more sub-movements to administer the task with certain accuracy. In 2007, Meng *et al.* surveyed four typical persons who simulated quadriplegics operating a trackball with their right dorsal hand and the kinematic parameters of cursor moving were measured [5]. The single subject experimental research (SSER) with alternating treatments design was used to compare the effects of four cursor moving direction (right to left, down to up, left to right, and up to down) on the kinematic variables. From analyzing the parameters of deviation from the straight line, velocity, movement unit and execution time, the efficiency to move on the horizontal direction (left to right or right to left) was better than moving in the vertical direction (up to down or down to up). When comparing the movement towards the left to the movement towards the right, participants required less execution time, velocity, movement unit and deviation from the straight line to position the cursor on leftward targets than in targets on the right.

Although the aforementioned studies showed the efficiency on the leftward direction compared with that of moving rightwards, the study of Dillen *et al.* did not reach this finding. Dillen *et al.* studied cursor trajectories controlled with a touchpad [6] and found targets to the right were reached faster than those to the middle or the left. Their findings showed movements to the right produced fewer numbers of submovements when compared with those to the middle or the left. The advantage of rightward movement while using a touchpad found by Dillen *et al.* is different from the leftward movement advantage found by the aforementioned studies. This can be explained that the fact that the demands of the required biomechanics of touchpad use are different from those of mouse control.

**Figure 1.**
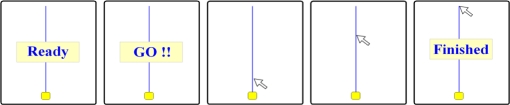
The cursor moving task procedure. Subjects were required to move the arrow tip of cursor starting from the yellow square, which is fixed in the start point, to the end of the straight line. This figure demonstrates the example of moving upward.

**Figure 2.**
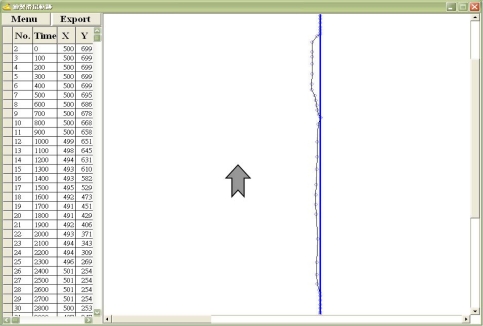
The graphical user interface (GUI) was used for displaying path and data coordinates during the cursor moving task of this study. In the right part of the screen, the blue line indicated the optimal trajectory of cursor moving and the black dotted line showed the subjects’ tracking path. The tracking path was sampled every 100 ms and the coordinates are displayed in the left table of the screen simultaneously during the upward moving task.

Several kinematic variables, including the initiation time (reaction time), movement time, total path of trajectory, velocity, and movement unit, were considered in understanding and indicating the changes of cursor moving [1–6]. Initiation time is defined as the latency between the display of the start signal and the beginning of the yellow square movement [10] (Figure 1). Movement time is defined as the time from the beginning of the yellow square movement to the end point when executing a moving action [1–6,10] (Figure 1). Total path is defined as the total length of the trajectory of the cursor dragged by subjects [1–4,10] (Figure 2). A movement unit is defined as one acceleration and deceleration phase [1–6,10]. More movement units represent worse control performance. Moreover, the power spectral density (PSD) of velocity is adopted to describe how the power of a time series is distributed with specific frequency. Using FFT we can convert the dragging velocity of single point to the frequency domain, which indicated power at the frequency during cursor moving. In this study, we explored the effect of direction on cursor moving as controlled by a regular mouse to accumulate more kinematic data for the purpose of clarifying the laterality issue when controlling cursors. Based on the aforementioned studies, we hypothesized that moving towards the left is more efficient from a kinematic point of view than moving in other directions.

## 2. Methods

### 2.1. Participants

The participants were 24 right handed healthy college students (12 males and 12 females; mean age: 20.13 years) without any neuromuscular or cerebral disease. The averaged handedness quotient of self reported Edinburgh handedness inventory was 93.96 (±5.71). These 24 students were also the same participants in the cursor dragging study conducted by us in 2007 [10].

### 2.2. Apparatus and Measuring Program

The cursor moving task was performed on a 1.8 GHz Pentium 4 laptop (ASUS, Taiwan) with a 14” XGA screen. A computer measurement program was developed to detect the real time kinematic characteristics during cursor movement and to provide the post processing of kinematic parameters after cursor movement. Participants used a standard 4D optical mouse (E. Sense, Taiwan) to move a cursor from four different home positions (*i.e.*, top, bottom, left, and right of the screen) to their opposite sites (see Figure 1 for an example of an upward movement). These four home positions were located 2.5 cm from the screen bottom, 0.8 cm from the top, 3.8 cm from the left and 6.5 cm from the right, respectively. Thus, the tracing lines were either horizontal or vertical, starting with the home positions and ending at a point 18.3 cm away, to match the trajectory. Participants were asked to move the cursor along the straight tracing line as close as possible. We have used this apparatus and measurement program in a group study to explore the effect of direction on dragging kinematics as controlled with a standard 4D optical mouse (E. Sense, Taiwan). The participants went from one point (home position) to the other point on the same line and did not return to the home position. They moved leftwards and rightwards from the point (839, 339) to (159, 340) and from the point (159, 340) to (839, 339), respectively. For the upwards and downwards movements, participants moved from the point (500, 699) to (500, 19) and from the point (500, 19) to (500, 699), respectively. Moreover, this apparatus and measurement program were also successfully used in a single subject experimental research design, to study the effect of direction on cursor moving kinematics with a trackball mouse, with significant improvement in the cursor reaching and stabilization on the toolbar area [5,8].

During the task, the measurement program sampled kinematic data every 100 ms. When the task was done, the moving trajectory and the log file would be exhibited on the screen to monitor the data simultaneously with participants’ performance (Figure 2). The log file contained the information of moving cursor position, which can be exported for further processing to determine the velocity of movement and other kinematic data. All statistical tests were two-tailed, with the significance level (*α*) being set at 0.05. All analyses were performed using SPSS version 10.0 (SPSS Inc., Chicago, IL, USA).

### 2.3. Experimental Design and Procedure

During the experiment, the participants sat on a fixed regular chair and table, with screen and mouse on it. The distance from the display to the edge of table as well as to the body of each participant was kept as similar as possible. During the tracking task, the participants used their right (dominant) hand to move the mouse in their most familiar and comfortable way. Each participant conducted two blocks of moving tasks (four directions each block). The order of four directions (left to right, right to left, up to down, and down to up) was pseudo-randomized and counterbalanced in each block across 24 participants. Therefore, the experiment included 24 different sequences (4! = 4 × 3 × 2 × 1) totally and each sequence was conducted by one participant (24 × 1 = 24). As the target area and the distance were the same in all directions, these two important factors that may confound the results were well controlled.

### 2.4. Statistics

Multiple one-way repeated measures ANOVAs were used to survey the effect of moving direction on different kinematic variables. Multiple *post hoc* LSD tests were conducted to compare the difference between two directions if the ANOVAs indicated a statistical significance. Based on the aforementioned interaction between velocity and time, the PSD figure was constructed to delineate the interaction between power and frequency among different moving directions.

## 3. Results

### 3.1. The Interaction between Velocity and Time Series among Four Directions

**Figure 3.**
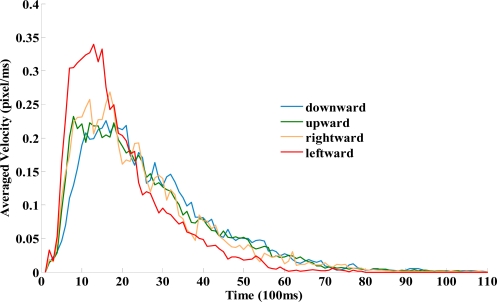
The interaction between velocity (pixel/ms) in each cursor moving direction.

Figure 3 demonstrated the velocity of cursor moving in four directions. The results indicated that the cursor moving velocities raised rapidly to their peak value in the first two seconds and then decline slowly to motionlessness in about 5 seconds. The averaged velocity data shows no significant difference in cursor moving with downward, upward, and rightward directions (Figure 3 and Table 2). The leftward cursor movement shows significantly higher average velocity (*p* < 0.01).

### 3.2. The One-Way Repeated Measures ANOVAs

The one-way repeated measures ANOVAs demonstrated that there were no significant differences across the four directions in reaction time (F(3, 69) = 0.092; *p* = 0.964; *Eta^2^* = 0.004), total path (F(1.731, 39.802) = 2.201; *p* = 0.130; *Eta^2^* = 0.087), and latency of peak velocity (F(2.184, 50.237) = 4.319; *p* = 0.016; *Eta^2^* = 0.158). However, the significant differences existed across the four directions in movement time (F(3, 69) = 15.121; *p* = 0.000; *Eta^2^* = 0.709), movement unit (F(3, 69) = 9.832; *p* = 0.000; *Eta^2^* = 0.299), average velocity (F(2.269, 52.180) = 18.654, *p* = 0.000; *Eta^2^* = 0.448) and peak velocity (F(3, 69) = 10.058; *p* = 0.000; *Eta^2^* = 0.304) (Table 2).

### 3.3. The Post Hoc LSD Tests

The mean values of each variable in each direction are listed in Table 3. *Post hoc* LSD tests (Table 3) showed that average velocity upward moving is higher than downward and leftward moving is higher than any of the other three directions. Fewer movement units and less movement time were found in upward and leftward moving when compared with downward moving and any other three direction movement. Regarding the peak velocity, moving upward is slower than the other three directions, and moving downward is slower than leftward.

### 3.4. The Figure of Velocity PSD

**Figure 4.**
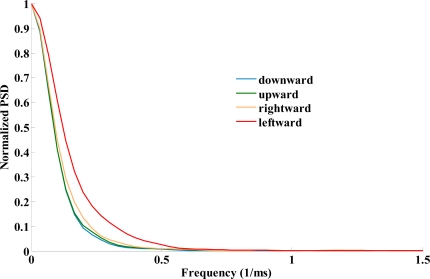
The PSD (power spectral density) of velocity.

Figure 4 shows the result of velocity PSD among the four cursor moving directions. The results indicated that the highest power in the lower band (<0.5 Hz) was observed in the direction of moving leftward in comparison with the other three directions. On the other hand, tight relationships among these four moving directors were occurred when the frequencies of cursor movements are greater than 1 Hz.

## 4. Discussion

The results of this study support the notion that kinematic parameters are influenced by cursor movement direction. When compared with moving rightwards, upwards and downwards, moving leftwards showed faster movement time, less movement units and better average velocity (Table 3 and Figure 3). Moving leftwards also showed larger peak velocity when compared with moving upwards and downwards. Consistently, those findings were also reported by [1–5] from 1998 to 2007. We noticed that all the participants in the aforementioned studies were right handed and were required to move a mouse (not to drag the mouse). Therefore, we infer that the best cursor moving efficiency occurred in the left direction in right handed persons while moving a mouse.

The directional effects reflect a superiority of the left movement for cursor moving in right handed people [2]. Morgan *et al.* mentioned that adductive movements (*i.e.*, leftwards) with a digital pen were of shorter duration and more accurate than abductive movements (*i.e.*, rightwards) [11]. Philips, *et al.* studied cursor trajectories as controlled by an accupoint and supported the biomechanical statement made by Morgan *et al.* Moreover, they emphasized that lateral movements involve only single joints and therefore were faster than vertical movements which involve multiple arm-segments [1]. However, the biomechanics of controlling a mouse in this study are different from those of controlling a digital pen and an accupoint. According to the task analysis done by us, it is not necessary to move the shoulder and elbow when the pisiform and lunate bones of the wrist are treated as the fulcrum on the table while moving a mouse horizontally. In fact, wrist radial deviation and ulnar deviation are the two major movements responsible for moving leftwards and rightwards, respectively. Furthermore, biomechanical knowledge indicated that a movement of the wrist toward the thumb side of the forearm (the radial deviation of wrist) has more degrees of freedom than the movement toward the little finger side (the ulnar deviation of wrist) [12]. The radial side of hand is also called the skilled side and most objects are manipulated on this side (few objects were manipulated on the ulnar side) [13]. Therefore, that moving leftwards showed better kinematic efficiency than moving towards other directions is reasonable.

The cursor moving task and related kinematic measurements used in this study might be further applied in clinical populations. Manto found the kinematic pattern of the discrepancy between centrifugal and centripetal movements in patients with psychogenic ataxia was unique when compared to healthy and cerebellar cortical atrophy subjects [14]. Therefore, Manto suggested that the analysis of movements in opposite directions might contribute to delineate the characteristics of some clinical populations or support the diagnosis of some populations, such as the comparison of centrifugal and centripetal movements in the vertical plane. Besides the potential application to patients with psychogenic ataxia, the cursor moving task in this study might help us to identify the kinematic differences between the leftward and rightward directions in patient with hemi-neglect. Mattingley, Bradshaw, and Phillips found directional hypokinesia is stronger in neglect patients with posterior cortical lesions [15]. They are determined to be particularly impaired in tasks requiring movement in a contralesional direction through measuring movement initiation and execution times for leftward and rightward movements in either hemispace and across the body midline. The cursor task in this study includes executing the movements towards the left and right in either hemispace and is thus potentially suitable to substantiate the clinical characteristics in patients with neglect syndrome.

On the other hand, the index of velocity PSD has been applied to study clients with multiple sclerosis and dysgraphia [16,17]. Longstaff and Heath found the rhythmic movements conducted by the healthy persons are more similar from trial to trial than those of the persons with multiple sclerosis who displayed tremors [16]. Smits-Engelsman and van Galen adopted the concept that higher velocity PSD means higher noise level to explain the results of clients with dysgraphia (poor writers) and their substantiation can be connected more directly to this study. They found that writing movements of poor writers’ are characterized by higher absolute noise levels for the higher frequency bands (5.5- and the 8-Hz) [17]. Conversely, good writers have relatively more power than the poor group in the lowest frequency bands (<2.5 Hz) under the same writing tasks [17]. Smits-Engelsman and van Galen further substantiated that more power in the lowest bands in good writers may play an important role to intermittent feedback for accuracy as well as to track visual targets for the purpose of rapidly positional corrections. In this study, the highest power in the lowest frequency band (<0.5 Hz) occurred in the direction of moving leftward and this situation might enhance accuracy and rapid positioning while moving leftward according to the statement by Smits-Engelsman and van Galen. In fact, moving a cursor towards the left was really the most efficient in this present study because the results demonstrated that the cursor movement of this direction achieved least movement units (most smooth) and best velocity.

Although the higher power of lower frequency might produce a larger force to make the cursor move, the results demonstrated that moving a cursor leftwards is more efficient in velocity and smoother in cursor control than moving towards other directions. However, the higher velocity may produce a larger force to make cursor movement less controllable. Why was moving the cursor leftwards still smoother (with fewer movement units) than moving towards other directions? The velocity PSD result may help us to explain that fact. The unique velocity PSD pattern (Figure 4) of the left direction showed that the higher power in lower frequency band provides the opportunity for subjects to adjust the movement performance compared with that of the other three directions. Therefore, moving the cursor leftwards was smoother, even working in the unstable situation with larger power of movement frequency.

## 5. Conclusions

The results of this study showed that moving leftwards is the most efficient from a kinematic point of view when compared with the other three directions. This finding is in agreement with experiments conducted by Phillips *et al*. [1–3], Thompson *et al*. [4] and Meng *et al.* [5]. Three studies [2–4] and this study employed right handed persons and used a regular mouse to operate the mouse for cursor moving tasks. Consequently, the directional effect on kinematic variables while using a regular mouse is consistent among right-handed adults. The navicular radial joint, the skill side of the hand which is compatible with the leftward cursor movement, may contribute to this phenomenon. Moreover, based on the aforementioned information obtained from healthy right-handed adults, the performance in persons with disabilities can be further studied for the purpose of substantiating their cursor kinematic characteristics and contributing to support the diagnosis of some populations, such as the patients with cerebellar cortical atrophy and with neglect syndrome researched by Manto *et al.* and Mattingly *et al.*, respectively [14,15]. Furthermore, the development of strategies to remediate or compensate for any disadvantages based on the cursor kinematic characteristics on each direction for persons with disabilities can be considered [3,5,8–10]. For example, if a patient demonstrates a disadvantage while moving towards the left, we can relocate the icons from the left side to the right side on the screen desktop, then the demands to move the cursor leftwards will be much less and this patient can perform more functionally. Therefore, potential guidelines for effective cursor movement can be formulated gradually for subjects with movement impairments [3,5,8,10] in the near future.

## Figures and Tables

**Table 1.** The effect of cursor moving direction on kinematics based on the literature review.

**Reference**	**No. of Participants (healthy adults)**	**Instrument**	**Kinematic variables with significant directional effect**	**Efficient direction**
[1]	12	Accupoint	Movement time, Movement unit	Horizontal (left and right)
[2]	12	Regular mouse	Movement time, Movement unit, Occurrence of overshooting	Left, up and down
[3]	12	Regular mouse	Movement time, Occurrence of overshooting,	Left
[4]	40	Regular mouse	Movement time, peak velocity	Horizontal (left and right)
[5]	4	Trackball Mouse controlled with right dorsal hand	Movement time, Total path, Velocity, Movement unit	Left
[6]	14	Touchpad	Movement time, Occurrence of overshooting, Submovement	Right

**Table 2.** The mean values of kinematic variables of each direction and the results of ANOVAs.

	Downward	Upward	Rightward	Leftward	*F* & *P* values	*Eta^2^*
Reaction Time (ms)	461.670	424.170	444.370	430.210	F(3, 69) = 0.092; p = 0.964	0.004
**Movement Time (ms)**	4,596.250	4,181.460	4,310.630	3,120.830	**F(3, 69) = 15.121; p = 0.000**	**0.709**
Total Path (pixel)	684.090	687.566	700.934	687.626	F(1.731, 39.802) = 2.201; p = 0.130	0.087
**Movement Unit**	11.690	10.440	11.020	8.190	**F(3, 69) = 9.832; p = 0.000**	**0.299**
**Average Velocity (pixel/ms)**	0.179	0.200	0.194	0.269	**F(2.269, 52.180) = 18.654; p = 0.000**	0.448
**Peak Velocity (pixel/ms)**	0.575	0.481	0.674	0.708	**F(3, 69) = 10.058; p = 0.000**	**0.304**
Latency of Peak Velocity (pixel/ms)	2,095.625	2,070.208	1,799.792	1,336.667	F(2.184, 50.237) = 4.319; p = 0.016	0.158

**Table 3.** LSD *post-hoc* tests.

	Average Velocity		Movement Unit		Movement Time		Peak Velocity	

	*MD*	*p*	*MD*	*p*	*MD*	*p*	*MD*	*p*
Downward *vs.* Upward	−0.021	**0.008**	1.250	**0.037**	414.792	**0.032**	0.094	**0.005**
Downward *vs.* Rightward	−0.015	**0.208**	0.667	**0.430**	285.625	**0.340**	−0.099	0.070
**Downward *vs.* Leftward**	−0.090	**0.000**	3.500	**0.000**	1475.417	**0.000**	−0.133	**0.007**
Upward *vs.* Rightward	0.007	**0.605**	−0.583	**0.451**	−129.167	**0.579**	−0.193	**0.001**
**Upward *vs.* Leftward**	−0.068	**0.000**	2.250	**0.000**	1060.625	**0.000**	−0.228	**0.000**
**Rightward *vs.* Leftward**	−0.075	**0.000**	2.833	**0.001**	1189.792	**0.000**	−0.035	0.510

MD: mean Difference.
